# Supplementary material for: Are carbon emissions trading and green financial instruments synergistic? -Comprehensive quantitative research based on content analysis
Source: PLoS One. 2024 Mar 7;19(3):e0298601. doi: 10.1371/journal.pone.0298601 (PMC10919601; doi:10.1371/journal.pone.0298601)
Supplement: S1 Data — (ZIP) [file pone.0298601.s001.zip › Data/地级市Z_F工作报告.zip!/╔╜╬≈/╨├╓▌╩╨/└┤╫╘╣1⁄2╓┌║┼í╛┬φ┐╦╩2╛▌═°í┐_2015.docx]

各位代表：

　　现在，我代表市人民政府向大会报告工作，请予审议，并请市政协委员和其他列席会议人员提出意见。

　　一、2014年工作回顾

　　过去的一年，面对严峻复杂的形势，我们坚决落实中央和省委、省政府决策部署，牢牢把握稳中求进总基调，积极适应经济发展新常态，在十分困难的情况下，立足忻州实际，努力做好工作，全市发展环境不断优化，群众生活得到改善，经济社会持续健康发展。

　　一年来，我们千方百计稳增长，坚定不移调结构。切实加强经济运行监测，针对性出台了促进经济发展的31个文件以及煤炭11条、工业5条等具体措施，着力破解突出困难问题，推动产业转型发展、企业转化升级。

　　坚持产业第一。煤炭现代化矿井改造建设步伐加快，煤电一体化积极推进，争取到3个总装机272万千瓦的“1920”低热值煤发电项目，一批风电、生物质能发电、煤层气发电、光伏发电项目取得实质性进展，新型综合能源基地已现雏形。铝工业循环经济园区纳入全省布局。煤机装备制造基地形成轮廓，法兰产业整合提升加快推进。节能环保产业快速发展。电子商务开始起步。芦芽山、雁门关创建国家5A级景区稳步实施，“又见五台山”成功上演。服务业占地区生产总值比重同比提高1.3个百分点。新兴产业投资占工业投资比重达到56.57%。

　　坚持项目至上。深入开展“项目见效年”活动，项目储备、签约、落地、开工、建设、投产年度任务全面完成。实行产业项目分类考核，119个重大产业项目成为项目建设的主力军。完善基础设施，保德至兴县瓦塘等铁路项目推进顺利，原神、繁五高速公路等交通设施项目建设加快。加大产业集聚区建设力度，提升“一园六企”水平。狠抓招商引资，发展“飞地经济”，区域协同、板块发展迈出新步伐。

　　坚持企业为重。清理规范涉煤收费项目，面向煤炭企业的省定行政事业性收费全部取消，违规收费项目全部取缔，保留收费项目全部规范；推进煤炭资源税从价计征改革；暂缓“两金”提取，吨煤降低成本45元。落实减税政策，为小微企业减免增值税、营业税、所得税5098万元。着力解决企业的融资难、融资贵问题，推广“助保贷”为146户企业落实贷款5.2亿元，通过政银企对接确定111个拟支持项目、资金59.19亿元。

　　坚持服务为本。新的市级政务大厅启动运行，56个审批部门入驻。主动公布政府部门权力清单，成为全省第一个推行权力清单制度的地级市。大力支持实体经济发展，市县两级财政累计投入中小企业发展资金1.22亿元，全市新创办小微企业1225户、新增“小升规”企业44户、新培育“小巨人”企业22户。

　　2014年，全市地区生产总值完成680.3亿元，同比增长5.4%；规模以上工业增加值308.4亿元，同比增长6.7%；全社会固定资产投资965.4亿元，同比增长18.4%；社会消费品零售总额275.1亿元，同比增长11.8%；外贸进出口总额20799万美元，同比增长5.3%；公共财政预算收入80.8亿元，同比增长9.6%；城镇居民人均可支配收入21735元、同比增长8.6%，农村居民人均可支配收入6104元、同比增长11.2%；居民消费价格涨幅1.9%，城镇登记失业率3.29%。

　　尽管受宏观经济增速放缓等各种因素影响，去年地区生产总值增速等部分指标未能达到预期，但经过全市上下的共同努力，在全省实现了较好排序。约束性指标全面完成，重要民生指标全部实现预期目标。

　　一年来，我们蹄疾步稳推改革，咬住重点促创新。坚持问题导向，通过改革释放发展红利，通过创新破解发展难题。

　　坚持综改统领。深入开展“转型综改攻坚年”活动，20项重大改革、30项重大事项、32个重大项目和3项重大课题等年度行动计划全面推进。切实转变政府职能，市政府机构改革全部完成，县级政府机构改革基本完成。深化行政审批制度改革，取消审批4项，调整审批53项，下放县级审批36项、承接省级下放审批33项。坚持一厂一策，运用“人资分离”等办法，实施了市钨丝厂等7户市属国企改革，解困职工2323人，其中置换身份1784人。落实煤焦公路销售体制改革部署，全部取消对相关企业的21项行政授权，全部取消煤焦公路运销9种票据，全市31个煤焦公路检查站点全部撤销。积极推进商事制度改革，实施“先照后证”，将工商登记113项前置审批改为后置，全面实行公司注册资本认缴登记制度，简化住所（经营场所）登记手续，改革市场主体年检验照制度，新登记注册私营企业同比增长66.18%。

　　坚持创新驱动。设立我市第一支私募股权投资基金，首期募集资金2.3亿元；鼓励民间资本进入金融领域，设立110家小额贷款公司，4家村镇银行、2家农商行共吸收民间资本17.1亿元。强化企业技术创新主体地位，累计培育国家级企业技术中心1家、省级企业技术中心15家、市级企业技术中心77家，认定高新技术企业20家，同脉冲式旋流澄清净水装置、环保型煤粉锅炉、无模密封件等一批技术创新成果成功实现产业化，成为忻州自己的科技品牌。

　　一年来，我们统筹城乡促发展，真心实意惠民生。始终把保障和改善民生作为一切工作的出发点和落脚点，尽力为群众办实事、办好事。

　　进一步改善城乡面貌。开展第三个“大干城建年”活动，总长57.44公里的城区“8+22”道路建设与改造工程全面竣工通车，云中河景区开园，3年新建改造道路总长116.21公里，城区建成区面积由28平方公里增加到71平方公里。岢岚等5个无管道燃气县具备通气条件，全市16座污水处理厂正常运行，12座生活垃圾卫生填埋场全部建成，城市（含县城）建成区绿化覆盖率增加4.5个百分点。保障性住房超额完成任务，建成了一批便民市场。全市城镇化率提高1.62个百分点，达到44.66%。实施改善农村人居环境4大工程，完成投资14.81亿元，改造县乡公路275公里，解决224个村8.8万农村人口和55所农村学校2.46万师生饮水安全问题，建成老年人日间照料中心100个，实施涉及2.95万人的采煤沉陷治理搬迁。全力办好农村“五件实事”，改造农村困难家庭危房11200户，新建改建农村幼儿园30所，易地搬迁农村贫困人口3万人，乡村清洁工程覆盖到行政村，培训新型职业农民8000名。

　　进一步做好“三农”工作。在持续推进“两增三建”工程的基础上，加快实施“四化五进”。坚持特色化、品牌化、规模化、市场化理念和主食化、副食化、礼品化、原料化方向，推动农副产品进城镇超市、进旅游景区、进贸易物流、进电商网购、进工业领域，全市农产品加工销售收入增长15%。忻州荣获“中国杂粮之都”称号，神池荣获“中国亚麻油籽之乡”称号，全市新增“三品一标”认证产品84个，全年羊饲养量达到700万只以上。农业综合生产能力不断增强，14座中小型病险水库除险加固工程完工，实灌面积达到212万亩，农业综合机械化水平达到60%，增长5个百分点。粮食总产35.4亿斤，创历史新高。产业扶贫扎实推进，8户省属企业落实扶贫项目13个，38户市域民营企业投入产业扶贫资金14.78亿元。全年减贫9.26万人。

　　进一步改善生态环境。狠抓节能减排，全市万元地区生产总值能耗降幅超额完成任务，提前2个月完成淘汰20万吨水泥、0.13万吨硅钙落后产能年度任务。排污权交易平台建设稳步推进。重点行业脱硫脱硝加快实施。淘汰黄标车和老旧车11757辆。忻州城区二级以上天数304天，同比增加17天。PM2.5和PM10年均浓度分别同比下降25.77%、13.68%。完成APEC峰会空气质量保障任务。深入开展“林业生态建设年”活动，认真落实“三加三不减”要求，完成营造林68.79万亩，财政林业投资同比增长66%。

　　进一步改善群众生活。全面加强社会保障，建立统一的城乡居民基本养老保险制度，把进城落户农民纳入社会保障体系。城镇居民医保和新农合年人均财政补助标准由280元提高到320元，工伤保险待遇标准提高10%，城乡低保标准每人每月分别提高25元、22元，农村五保对象集中供养、分散供养补助标准每人每年分别提高200元、130元。对7231名重度残疾人和贫困残疾人进行补贴，对5000名贫困残疾人进行康复救助，为23051名困难家庭学前幼儿提供生活补助。千方百计扩大就业，制定实施20多项促进就业的扶助政策，重点扶持高校毕业生创业和小微企业吸纳劳动者就业，促进农村劳动力转移就业，推动创业带动就业。实行失业保险市级统筹，重视破产改制企业职工安置工作，保障失业人员基本生活。全市城镇新增就业3.72万人，转移农村劳动力3.84万人，创业就业5400人，公益性岗位安置就业困难高校毕业生1148人。着力增加居民收入，提高最低工资标准和企业工资增长指导线，为全市8.2万名企业退休人员提高基本养老金，月人均增加221元；提高市直机关津补贴标准和事业单位绩效工资水平，并向低职务职称人员倾斜；为领取失业保险金人员发放冬季取暖补贴，月人均480元；为全市农户免费发放98万吨取暖用煤；建立健全治理农民工欠薪工作机制，为5518名农民工追讨工资4858万元。认真做好物价工作，加强与群众生活密切相关的房地产、水、暖、电、气等商品价格以及医疗卫生服务价格、学校及幼儿园教育收费等重点领域价格监督管理，开展“一元菜店、平价商店”活动，实现了惠民价稳。

　　进一步满足群众需求。新改扩建公办标准化幼儿园15所，改造农村幼儿园30所；推进义务教育学校标准化建设和均衡发展，启动二期农村义务教育“全面改薄”，义务教育招生及均衡编班得到规范；落实边远贫困地区教师待遇，招聘269名农村义务教育特岗教师，选派130名教师到贫困县支教，建立校长教师交流制度，共交流教师2731名、校长106名；中职教育免收学费全覆盖惠及29414名学生，普通高中学生助学金受助学生12001人。深化医药卫生体制改革，在8个县实施县级公立医院综合改革，开展非政府办基层医疗卫生机构实施基本药物制度试点工作，人均基本公共卫生服务经费财政补助标准提高到35元，城乡居民大病保险率先在全省开始报销结算，参合农民住院按病种分级诊疗试点平稳起步，273户家庭享受到“单独两孩”政策，新建市人民医院竣工。加快发展文化事业，“周末大戏台”“送戏下乡”、农村公益电影放映等文化惠民工程扎实开展，第四届“梨花奖”舞台艺术大赛成功举办，文化产业整体实力有了新的提升，文物、非遗保护得到加强，群众文化活动多姿多彩。承办了全国中国式摔跤搏克挠羊冠军赛等一批赛事，我市体育健儿在第14届省运会上取得好成绩。

　　一年来，我们毫不放松抓安全，全力以赴保稳定。始终把安全生产作为一条不可逾越的红线，把维护人民群众生命财产安全作为最重要的责任，努力创造和谐稳定的发展环境。

　　全面落实中央和我省加强安全生产的一系列部署，坚持党政同责、一岗双责、齐抓共管。深入实施“443”工作机制，严格落实两个“主体责任”，以铁的纪律推动各级各部门各企业“知责、履责、尽责”。突出抓好煤矿、非煤矿山、危险化学品、油气管道、交通运输、隧道交通等重点行业领域安全生产，扎实开展“六打六治”打非治违行动，持续引深安全生产大检查，不断夯实安全生产基层基础，着力消除安全生产隐患和薄弱环节。全年未发生较大以上安全生产事故，煤矿百万吨死亡率0.053，安全生产形势持续稳定好转。

　　进一步加强和创新社会治理。着力抓好矛盾纠纷排查和源头预防，重视解决农民工欠薪和非法集资问题，充分发挥社会力量在信访工作中的重要作用。加强社会治安管控，在全省率先推行PTU、SDU新型巡逻防控模式，严厉打击各类违法犯罪行为，扎实推行城镇社区网格化管理，积极探索农村社区管理新模式，充分发挥人民调解员作用，群众安全感不断增强，打造山西最安全城市迈出新步伐。

　　一年来，我们从严治政守底线，接受监督转作风。紧紧依靠群众，严格依法办事，加强自身建设。

　　加强民主法制建设，认真执行市人大及其常委会的决议、决定，积极支持人民政协履行职能，全年办理市人大代表建议163件、市政协委员提案355件，全部办结。支持各民主党派、工商联、无党派人士参政议政，支持工会、共青团、妇联等人民团体发挥作用。认真做好行政复议工作，切实维护群众合法权益。深入开展“六五”普法，公民法治意识进一步增强。

　　自觉接受人大法律监督、政协民主监督、纪检部门的执纪监督和社会舆论监督。建立“呵护美丽忻州随手拍”微信问政平台和“忻州发布”政务微博，回应群众关切，改进工作不足，“随手拍”被中央网信办评为优秀政务公众账号，“忻州发布”位居人民网2014年全国政务微博办实事总排行榜前列。

　　坚持不懈反“四风”、转作风。深入开展政府系统党风廉政建设和反腐败斗争，严肃查处领导干部违纪违法问题和重点领域腐败案件，廉政风险防控进一步加强。严格执行中央“八项规定”精神、国务院“约法三章”，严控“三公”经费支出，市、县两级“三公”预算全部公开。

　　各位代表！一年来，我们牢记宗旨，保持定力，戮力同心，攻坚克难，取得了忻州经济社会发展来之不易的新成绩。这些成绩的取得，靠的是党的坚强领导和人民群众的努力奋斗，靠的是人大、政协的大力支持和有效监督，靠的是各方面的上下齐心、众志成城。在此，我代表市人民政府，向全市人民、驻忻部队指战员、武警官兵、公安民警和各驻忻单位，向各民主党派和人民团体，向所有关心、支持忻州发展进步的朋友们，致以崇高的敬意，表示衷心的感谢！

　　我们清醒地认识到，我市经济社会发展仍然存在很多困难和问题。经济下行压力持续加大，企业运行困难，效益大幅下滑，市场信心不足，稳增长任务艰巨；长期积累的经济结构不优、质量效益不高、资源型经济比重较高的问题仍未得到显著改善；国企改革任务艰巨，民营经济发展滞后，创业创新氛围不浓；城乡居民收入较低，民生社会事业欠账较多，农村贫困落后的局面还没有得到彻底改变；安全生产基础薄弱，压力依然较大；群众关注的热点问题未能很好解决，维护社会和谐稳定任务繁重；政府作风建设与群众期盼还有差距，党风廉政建设和反腐败斗争形势严峻。

　　二、2015年工作安排

　　2015年，是全面深化改革的关键之年，是全面推进依法治国的开局之年，是全面完成“十二五”规划的收官之年，是我市全面推进“六大发展”的启动之年。做好今年的政府工作，要深入贯彻党的十八大和十八届三中、四中全会精神，认真落实省委十届六次全会和市委三届六次全会部署，以邓小平理论、“三个代表”重要思想、科学发展观为指导，以习近平总书记系列重要讲话精神为根本指针，按照“四个全面”战略布局，深刻认识、主动适应、积极引领经济发展新常态，坚持稳中求进工作总基调，坚持“产业第一、项目至上、企业为重、服务为本”，以提高经济发展质量和效益为中心，突出创新驱动，强化防控风险，把转方式调结构放到更加重要位置，全力推进“六大发展”，全面深化改革，扩大开放，保障改善民生，促进全市经济平稳健康发展，社会和谐稳定。

　　全市经济社会发展的主要预期目标是：地区生产总值增长6%左右，全社会固定资产投资增长16 %左右，社会消费品零售总额增长10%，地方公共财政预算收入负增长9%左右，城镇居民人均可支配收入增长7%以上，农民人均纯收入增长8%以上，外贸进出口总额增长3%，城镇登记失业率控制在4.2%以内，居民消费价格指数控制在3%左右。

　　约束性指标是：万元地区生产总值能耗下降3%，万元地区生产总值二氧化碳排放量下降3%，二氧化硫、化学需氧量、氨氮、氮氧化物、烟尘、粉尘排放量分别下降1.86%、1.52%、2.56%、9.04%、0.25%、0.5%，万元工业增加值用水量下降3.5%。

　　重点做好以下8个方面的工作：

　　（一）全面深化改革，推动创新创业，不断激发发展的动力和活力。

　　抓好重点改革。实施转型综改“1333”年度行动计划，确保3年任务全面完成。深化煤炭管理体制改革，继续落实煤焦公路销售体制改革举措，深化煤炭销售体制改革，加快煤炭项目审批和证照体制改革，巩固涉煤清费成果，做好煤炭资源税从价计征改革工作，促进全市煤炭工业可持续发展。深化企业改革，稳步推进云马焦化、忻州酒厂等市属国有企业改革，推动云河集团片区开发，巩固水务公司改革成果，加紧完成企业社会职能移交，认真谋划停产、半停产和不具备整体改制条件企业闲置资产盘活和职工身份置换问题，启动市属国有企业财务等重大信息公开；推动民营企业建立现代企业制度，加快建设管理、研发、营销团队。全面开展农村土地承包经营权确权登记颁证工作，有序推进农村土地流转；探索农村土地承包经营权融资改革，开展农村土地承包经营抵押贷款试点工作。全面启动不动产登记。

　　实施创新驱动。确立企业技术创新的主体地位，加强企业技术中心建设，支持企业建立研发机构，组建煤机制造、法兰锻造、铁矿采选行业技术联盟，推进创新成果产业化应用。积极培育扶持一批创新型企业，推动全市规模以上企业一户联系一所省级以上科研院所。实施高新技术企业育种工程，加快培育创新能力强、成长性好、具有一定市场竞争力的科技型企业群。深入开展科学技术奖评比活动，营造科技创新浓厚氛围。推进产学研联合，促进科技成果转化成现实生产力。认真开展企业“五小”活动，积极鼓励岗位创新和创造。深入开展万众创新活动，大力加强乡土科技人才队伍建设。抓好机制创新，千方百计增加科技投入。坚持探索创新，认真解决科技工作中存在的困难和问题。积极推进管理创新，增强企业的自我约束、自我管理、自我发展的能力。引导科技人员自强自立、敢为人先，增强企业专利保护意识和能力，推动有效发明专利拥有量稳步增长。

　　推进金融振兴。紧紧围绕信用建设、杠杆运用、风险防范三个环节，促进金融和实体经济的互利共赢、共同发展。积极培育合格的市场主体，不断优化金融生态环境。推动企业完善资产凭证、规范财务管理、完善法人治理结构、提升经营效益、搞好抵押物储备、聘用优秀管理团队，不断提升企业的信用等级；完善信用体系，加快建立统一的社会信用平台，开展信用县、乡、村、企业创建活动，开展公职人员逾期贷款清收专项整治，着力解决不诚不信的突出问题。深化金融改革创新，不断改善融资服务。深化政银企合作，引导金融机构不断创新服务，拓展信贷市场，着力支持市场前景好、符合产业政策、经理层有能力的企业。大力发展地方金融机构，年内引入1家股份制商业银行，增设5家县级支行、5家社区银行，新设宁武、静乐两家村镇银行，完成繁峙信用联社改制，启动代县、定襄信用联社改制。加快发展多层次资本市场，做好对全市51户拟上市企业的培训引导工作；加快推进股权融资，尽快推动一批企业在山西股权交易中心挂牌。努力扩大债券融资，积极运用创投基金、产业基金、私募基金等新的融资手段，放大“助保贷”成效，探索开展“税易贷”，积极稳妥推出新的特色金融产品。依法有序发展担保机构，做实政策性融资担保公司，鼓励民营资本参股融资担保公司，推动市县两级政府参股省再担保公司。提升小额贷款公司经营质量，盘活民间资本，规范民间借贷行为。积极发展会计事务所、律师事务所、审计事务所以及农村产权评估机构等金融要素市场，积极稳妥发展互联网金融。认真防范金融机构自身风险、贷款企业经营管理风险、恶意逃废银行债务、非法集资等金融风险，努力促进金融和实体经济健康发展。

　　倡导全民创业。毫不动摇地鼓励、支持、引导非公有制经济发展，注重发挥企业家才能，切实增强各类所有制经济活力。认真落实减轻企业负担“60条”措施，在“减、免、缓，帮、扶、替”上下功夫，促进企业轻装前行，扶持企业做大做强。进一步强化全民创业政策支持，深化商事制度改革，为创业企业工商注册提供便利。积极搭建创业平台，鼓励和扶持科技人才、外地务工回乡人员、大学毕业生、进城农民、下岗职工等重点人群创业。从今年起，市财政每年安排3000万元全民创业专项扶持资金，为各类创业主体提供创业贷款贴息、创业担保风险补偿、融资保费补贴及创业奖励补贴；安排3000万元中小企业发展专项资金，用于中小企业孵化基地、大学生创业园等集聚区建设。坚决清理不合理规定，消除各种隐性壁垒，帮助民营企业解决创办、创新、税费、融资、审批等方面的实际问题，让民营经济依法平等使用生产要素，公平参与市场竞争，同等受到法律保护，努力形成大众创业、万众创新的生动局面。

　　（二）转变发展方式，调整产业结构，做好煤炭和非煤产业两篇大文章。

　　加快综合能源基地建设步伐。落实煤炭产业“六型”转变要求，大力发展非煤能源，促进高碳资源低碳发展、黑色煤炭绿色发展、资源型产业循环发展。巩固和拓展煤炭市场份额，加快煤矿标准化建设，实现煤炭产量适度增长。加快煤电一体化，全力推进神华神东河曲2×35万千瓦、忻州广宇二期2×35万千瓦、晋能保德2×66万千瓦、华润宁武2×35万千瓦、山煤河曲2×35万千瓦、同华轩岗二期2×66万千瓦等一批低热值煤和燃煤发电项目。加快天然气和煤层气产业发展，推进晋西北天然气公司神池、五寨、偏关城市输气管网、保德—河曲区块地面煤层气产业开发和一批加气站项目工程建设。继续抓好风电、光伏发电等新能源产业。持续推进五寨400万吨煤制油联产20亿立方煤制天然气项目，推进煤矸石、焦炉煤气、煤焦油等循环利用，变废为宝。

　　加快资源型经济转型步伐。推进铝工业向新材料延伸，加快中电投山西铝业和同德铝业主产业链项目申请核准工作。优化同德铝业股权设置并尽快复工建设。优化装备制造业，加快定襄法兰锻造业兼并重组、整合提升，抓好忻府区晨辉机械制造、五台德奥电梯等项目。推动“忻材忻用、忻用忻材”，抓好“两型”产品使用推广。继续推进五台山改造提升，加快芦芽山、雁门关创建5A景区工作，提升景区整体功能和服务档次。抓住五台山机场即将建成投运机遇，注重市场开发，拓展旅游客源。加强文旅互动，不断开发体现地方特色的文化旅游产品。加强旅游市场管理，提升旅游服务质量。壮大节能环保产业，抓好蓝天锅炉、久力微晶石复合肥、特瑞环保除尘设备、道生鑫宇焦炉气制LNG等项目，把节能环保产业打造成我市重要的新兴支柱产业。发展物流商贸及现代服务业，稳定煤炭物流产业，加快永旺物流、豪德汇通商贸物流、忻州汽贸文化园等项目建设，推进文化体育、健康养老、家政服务等服务业发展，切实提高服务业占全市GDP的比重。扶持发展食品医药产业，立足我市农畜产品和生物制药资源优势，深化开发利用，推出拳头产品。按照“市场要活、创新要实、政策要宽”要求，不断发现和培育新的增长点。

　　加快集聚发展步伐。建设“三大门户”、打造“三大集散地”、发展“六大板块”，推动各县（市、区）优势互补，集聚发展。支持忻州经济开发区加快发展，切实发挥示范带动作用。推动“一县多园”“一县一园”“飞地经济”“一园六企”建设，鼓励符合条件的产业园区升级省级开发区。加快集聚区生产生活服务业发展，推动物流、仓储、金融、通讯、电子商务等生产性服务业配套。大力培育和发展商会、行业协会、咨询公司等市场中介组织，为集聚区发展提供服务。

　　（三）引深项目攻坚，搞好招商引资，努力促进经济平稳健康发展。

　　深入开展“项目提质增效年”活动。自觉把“扩量、加速、提质、增效”作为项目建设的主攻方向，贯穿项目建设的全过程、各方面，坚决不让低质低效项目进入储备盘子，严格新上项目用地审核，加强项目资金使用效益、单位土地产出效益以及节能节水、污染减排等约束性指标考核，在保障规模的基础上，加快资源节约型、环境友好型项目建设，把全市项目建设推向注重质量效益的新阶段。

　　持续加大投资力度。狠抓省市重点工程建设，项目储备投资总额动态保持9247亿元，项目签约投资总额1534亿元，项目落地投资总额873亿元，项目开工投资总额679亿元，重点工程完成投资812亿元，项目投产总投资712亿元。狠抓分类考核新开工产业项目，对去年项目跟踪问效，今年抓好总投资1431.54亿元的99个项目。狠抓10大重点领域投资，完成投资506.6亿元。其中：铁路投资21.06亿元，重点推进大西高铁忻州段、静游地方铁路、保德至兴县瓦塘铁路一期工程瓦塘至冯家川段等项目；公路投资52.67亿元，重点推进原神高速公路、晋蒙黄河大桥、神岢高速公路、208国道城区改线等公路建设项目；水利投资25.47亿元，重点抓好大水网骨干及县域供水工程、农田水利建设和河流治理工程等项目；外送电通道和电网建设投资20.84亿元，重点抓好途经我市的4条外送电通道建设和一批输变电工程项目；低热值煤发电项目投资30.3亿元；煤层气投资8.3亿元；节能环保投资3.8亿元；新兴产业投资219.8亿元；城乡人居环境改善投资126.36亿元。通过抓好这些项目，实现全年固定资产投资突破千亿元的目标。

　　严格落实项目责任。落实政府推进主体责任、项目单位实施主体责任、职能部门服务主体责任，把责任细化到每个环节、每个岗位和每个人头上，形成“九牛爬坡、个个出力”的局面。实行县（市、区）长联系项目制度，每名县（市、区）长负责联系抓好10个项目，发挥领导带头作用。继续坚持“八位一体”推进机制，完善项目台账管理信息平台、24小时直通车制度和重点工程实时跟踪服务制度，真正在改进服务上下功夫。

　　不断提升对外开放和招商引资水平。加快建设对外开放的“三个门户”，深化区域经济交流合作，积极融入京津冀和环渤海经济圈，主动对接“一路一带”，加大承接长三角、珠三角地区产业转移力度。围绕“336”布局和重点产业，抓好优势产业上下游产业链的延链、补链项目招商。继续坚持组团招商、展会招商、小分队招商，积极推动商会招商、企业招商、以商招商，探索开展驻点招商、委托招商、点对点招商，实现精准招商。突出差异化发展、个性化特色，切实增强招商引资政策的吸引力，以实际行动赢得客商的信赖和认可，努力把忻州打造成“近者悦、远者来”的创业乐土和投资洼地。

　　（四）发展现代农业，加快扶贫开发，推动农业稳定发展农民持续增收。

　　合理调整农业内部结构，集中发展主导产业。充分发挥“中国杂粮之都”“中国亚麻籽之乡”“中国红芸豆之乡”“中国藜麦之乡”等品牌优势，全面实施小杂粮“五个一”工程，推进农产品“四化五进”行动，精心打造“豆面、莜面、荞面、马铃薯、亚麻油、糯玉米、羊肉、月饼、毛健茶”等主食化、副食化、礼品化地方特色名优产品，年内新增认证无公害绿色有机和地标农产品50个以上，力争打造2个以上“国字号”优质名牌产品，推动相关产品商标和地理标志注册。扎实推进新一轮雁门关生态畜牧经济区建设，发展种草养畜，羊饲养量达到1000万只，推动恒兴、紫云牧业等屠宰企业建成投产，五寨绒毛加工企业和岢岚羊皮加工企业落地。继续抓好设施农业、发展“一县一业”“一村一品”。大力培育现代流通方式和新型流通业态，扶持发展特色农产品网上营销，推动我市优势小杂粮和畜产品占领更广阔市场。

　　加强农业水利设施建设。完成静乐东碾河、朱家川河保德段、偏关河河道治理续建任务，完成牧马河忻府段、峨河繁峙段改造工程主体任务，完成五台唐家湾水库应急除险加固工程，完成牧马河灌区节水配套改造工程续建任务，争取原平永兴河灌区节水配套改造工程开工。加快推进忻州城郊排洪应急工程和南云中河延伸工程，争取年内完成主体工程建设任务。

　　加快转变农业发展方式。大力推广节肥、节药、节水和清洁生产技术，推动农业废弃物转化处理，秸秆、畜禽粪便资源化利用和农田残膜回收。加强农业科技创新和集成示范，加快构建适应现代农业发展要求的技术体系，着力解决农技推广“最后一公里”的问题。大力发展乡村旅游和生态、观光、采摘、休闲农业，打造特色鲜明的乡村旅游产品。引导鼓励城市工商资本到农村发展适合企业化经营的种养业和农产品加工业，以工业化理念走好农业产业化路子。

　　坚持多措并举，加快整体脱贫步伐。认真落实精准扶贫开发机制，切实做到项目规划、帮扶措施、帮扶干部、扶持资金到村到户。继续推进易地扶贫搬迁工作，移民搬迁3万人，完成“十二五”规划的17万人的移民搬迁任务。继续推进百企千村产业扶贫开发工程，促进产业扶贫开发省属企业项目尽快建成投产、民营企业项目达产达效。加快以整村推进、彩票公益金项目为主的产业开发项目的实施进度。继续扩大教育扶贫规模，对贫困地区农村贫困大学生资助做到全覆盖，落实好国家重点院校对贫困地区大学生的录取优惠政策。确保全年减贫人口10万人，贫困县农村常住居民人均可支配收入增长高于全市平均水平。

　　（五）改善城乡面貌，加强环境保护，着力打造宜居宜业、天蓝水净的美好家园。

　　启动城市人居环境改善工程。把2015年确定为“城市建设管理提升年”，实施设施提升、城市安居、城中村改造、环境提质4大工程。中心城区围绕完善干线路网、改造小街小巷、配套便民设施、加大生态建设、强化城市管理目标，进一步完善基础设施，提升城市管理服务水平。完善干线路网，改造小街小巷：改造新建路、利民街、学府街、花园街等30条主次干道，形成完备的干线路网架构，进一步畅通微循环。配套便民设施：按照创卫标准，再建一批游园、绿地、便民市场、停车场，新建和改造一批地坑式垃圾中转站、公厕等便民配套设施；完成大西高铁站前广场、雁门广场建设。加大生态建设：围绕创建省级园林城市目标，启动九龙岗森林公园和云中河景区二期工程，进一步加大绿量、提高质量，城区力争新增绿地面积75万平方米。强化城市管理：充分发挥数字化城管“一个中心、四个平台”作用，进一步创新管理模式，加大管理力度，形成事件发现、快速处置、巡查督查、责任落实一体化高效运行的长效管理机制。启动中心城区和偏关县城“创卫”工作，巩固扩大“集成创卫”成效，深入开展城乡爱国卫生清洁运动，积极开展“卫生单位”、“卫生家庭”创建活动。以原平市区、保德县城和忻府区奇村镇等13个示范镇为重点，加快市政公用设施、公共服务设施和生态环境建设，提升“大县城”建设水平。新开工建设保障性住房24775套，其中城市棚户区改造21575套、公租房2118套、国有工矿棚户区改造1082户，基本建成10100套。加快实施城中村改造。落实放宽户口迁移政策，统一城乡户口登记制度。全面实施居住证制度，对在城镇就业居住但未落户的外来人口，以居住证为载体提供相应的基本公共服务。

　　加快推进农村人居环境改善工程。实施好完善提质、农民安居、环境整治、宜居示范4大工程，改善6.68万农村人口和7所农村学校2670名师生的饮水条件，绿化村庄60个，新建老年人日间照料中心100个，实施11.9万户广播电视卫星户户通提质工程；加快农村采煤沉陷区治理，完成搬迁安置10946户；完成36个村的污水处理任务，全面开展农村生活垃圾专项治理；抓好20个省市两级美丽宜居示范村创建。扎实办好“五件实事”，再改造农村困难家庭危房7650户，新建改建农村幼儿园32所，完成农村贫困人口易地搬迁任务，深入推进乡村清洁工程，培训新型职业农民12000人。

　　切实搞好生态建设。严格落实“三加三不减”要求，继续开展“林业生态建设年”活动，完成营造林72万亩和市级重点工程任务。全力做好全省造林绿化忻州现场会准备工作，努力出精品、出经验、出精神。继续深化集体林权制度改革，开展集体林权流转试点，推进林业专业合作组织建设，全面推进森林保险，积极推进林权抵押贷款。持续加大森林防火力度，牢固构建责任体系、防范体系、扑救体系、保障体系，确保不发生重大森林火灾。

　　强力推进节能减排。继续实行用电量与GDP挂钩和能源总量控制考核办法，严格控制矿热炉、精炼炉、电炉等高能耗项目的增容报装，全面推进能源消费量交易，开展企业能效对标活动。统筹推进工业、建筑、交通、公共机构等领域节能降耗，做好煤炭、焦化、冶金、电力、化工、建材等重点行业节能工作，完成淘汰落后产能任务。全面推进污染减排和大气污染防治工作，突出“控煤、治污、管车、降尘”关键环节，深入开展重点行业脱硫、脱硝、除尘改造，加强畜禽养殖污染治理，积极推广清洁能源，认真开展淘汰黄标车、老旧车工作。完成市污水处理厂中水回用、扩容提标改造工程。认真贯彻新的《环保法》，全面开展环境保护大检查，严厉查处各种环境违法行为。加强黄河、汾河、滹沱河等重点流域环境监管，加强水资源管理，用最严格的力度落实好“三条红线”制度。

　　（六）发展社会事业，保障改善民生，让发展成果更多惠及群众。

　　推动各类教育协调发展。大力发展学前教育，新建、改扩建13所标准化公办幼儿园。促进义务教育均衡发展，做好免试就近入学工作，推进薄弱学校改造和学校标准化建设。启动普通高中办学标准化建设，提升高中办学水平。发展现代职业教育，抓好各县职教中心和市职业技术学院实训基地建设。支持特殊教育发展，提高残疾儿童少年义务教育普及水平。规范中小学管理，加强教师队伍建设，加大城乡教师交流、校长轮岗力度。优化中心城区学校布局，完成忻州一中北校区、长征小学、实验幼儿园主体工程，服务好忻州师院新校区建设工作。

　　提升医疗卫生服务水平。深化医药卫生体制改革，用好改革试点经验，全面推开县级公立医院综合改革工作。继续加大基本公共卫生服务经费财政补助，人均补助标准提高到40元。完善城乡居民大病保险制度，加快发展商业健康保险。进一步完善基本药物制度，做好常用低价药品保障工作。积极推进分级诊疗制度，引导优质资源下沉到基层，鼓励社会资本办医，推进医师多点执业。加强村医队伍建设，改善乡村卫生医疗条件，提升基层医疗卫生机构服务水平。加快市人民医院新址功能完善，抓好市儿童医院、早教中心、残疾人康复中心建设。认真做好计生工作。

　　着力稳定和扩大就业。统筹抓好高校毕业生、农村转移劳动力、城镇困难人员、转业军人等各类群体的就业工作。大力发展劳动密集型企业，鼓励小微企业吸纳就业。加强政府公益性岗位就业管理，整合开发更多就业岗位。实施创业引领和促进就业计划，以全面创业带动全民就业。强化就业指导服务，搭建就业供需对接平台。精心做好残疾人、零就业家庭等就业困难人员的就业援助工作。实行困难企业缓缴社会保险费办法，并给予社保、岗位和培训补贴，鼓励支持企业不裁员或少裁员，稳定现有就业。

　　努力促进城乡居民增收。健全企业工资决定和正常增长机制，依法推进企业工资集体协商。执行省定最低工资标准，适时发布年度企业工资增长指导线。完善工资制度，提高机关事业单位基本工资标准并建立正常调整和增长机制，实行乡镇工作补贴，向条件艰苦的偏远乡镇和长期在乡镇工作的人员倾斜。落实县以下机关公务员职务职级并行制度。健全农民工工资支付保障机制，建立并用好农民工工资周转金，严厉打击恶意拖欠农民工工资行为，坚决保障农民工合法权益。

　　提高社会保障水平。落实机关事业单位养老保险改革措施，做好失业、工伤、生育保险工作，进一步完善覆盖城乡的社会保障体系。巩固并轨后的城乡居民养老保险制度成果，继续提高标准，企业退休人员基本养老金提高10%，城乡居民基础养老金每人每月增加15元。推进城乡居民基本医疗保险制度整合，城镇居民基本医保和新农合年人均财政补助标准由320元提高到380元。继续提高城乡低保、农村五保对象补助水平。做好残疾人就业援助、康复救助，全面实施临时救助制度。

　　支持国防和军队建设，做好双拥、优抚安置和人防工作。发展妇女儿童、老龄和红十字会、残疾人等事业。做好民族宗教、外事侨务等工作。加强气象、地震、科普、档案、史志等工作。

　　（七）加强文化建设，提升群众素质，凝聚和传播奋勇争先的强大正能量。

　　培育和践行社会主义核心价值观。大力弘扬改革创新的时代精神，充分发挥法治文化、红色文化、廉政文化资政育人的作用，深入开展社会公德、职业道德、家庭美德和个人品德教育。组织开展好纪念抗日战争暨反法西斯战争胜利70周年活动。倡导全民阅读。做好扫黄打非和净化网络工作。加强对外宣传和文化交流，讲好忻州故事，提高我市文化传播力和影响力，塑造忻州新形象，集聚推动发展的正能量。

　　完善公共文化服务体系。巩固经营性文化单位转企改制成果，支持转企改制国有文艺院团改革发展，推进公益性文化事业单位改革。加快文化基础设施建设，推进市博物档案馆、科技图书中心、忻州大剧院等重点工程，搞好县级“三馆一院”建设。认真抓好政府购买公共文化服务，继续实施“文化惠民”工程，精心组织“周末大戏台”“乡村大舞台”“梨花奖”舞台艺术大赛，完成10万户的广播影视“户户通“任务。鼓励精品创作，繁荣文化发展。加强文物保护工作。深入开展全民健身运动，进一步提高竞技体育水平。

　　加快文化产业发展。完善促进文化产业发展各项政策，支持骨干文化企业加快发展，促进金融资本投向文化产业。以发展文化创意产业为依托，努力为文化产业、文化产品和文化服务搭建平台，加快发展数字出版、动漫游戏、移动多媒体等新兴文化产业。积极参加各类文化产业博览交易会，精心搞好对外宣传，加大文化产业的招商引资力度。强化文化市场执法监管，切实维护文化市场秩序。

　　（八）狠抓安全生产，搞好社会治理，创造稳定和谐的发展环境。

　　持之以恒抓好安全生产。牢固树立安全发展的理念，牢记“三个决不能过高估计”，始终保持“三个敬畏”，牢牢掌握安全生产工作的主动权。全面落实新的《安全生产法》，扎实推进依法治安。坚持“党政同责、一岗双责、齐抓共管”，以开展“企业主体责任年”活动为契机，深入落实安全生产“443”机制，以政府监管责任的落实推动企业主体责任的落实。继续引深安全生产大检查行动，全面加强煤矿、道路交通、非煤矿山和尾矿库、危化品、人员密集场所以及建筑施工、油气管道、城市燃气、特种设备等各行业各领域安全隐患排查整治，始终保持安全生产的高压态势。强化食品药品安全监管，确保人民群众饮食安全、用药安全。支持消费维权，营造安全放心的消费环境。加强应急管理和防灾减灾能力建设，认真做好消防工作。强化考核问责，严格落实安全生产目标责任考核“一票否决制”，严肃查处事故，严格追究责任，坚决遏制重特大事故、减少一般性事故，不断向持续稳定好转的目标迈进。

　　加强和创新社会治理。推动政府治理和社会自我调节、居民自治良性互动，加强社区工作，强化市、县、乡、村四级服务管理（指导）中心职能，推进网格化管理，完善调处化解矛盾纠纷综合机制。把信访纳入法治轨道，及时就地解决群众合理诉求。加强社会治安综合治理，深入开展“打黑除恶”斗争和突出问题整治行动，严厉打击处理各类违法犯罪行为，防范和处置突发性群体事件，防止暴力恐怖事件发生。扎实开展基层平安创建活动，进一步完善治安防控体系。加强社区戒毒工作，不断净化社会环境。强化网络管理，坚决打击网上造谣、传谣行为，切实维护网络秩序，净化网络空间，决不允许网络成为法外之地。健全普法教育机制，落实“谁执法，谁普法”机制，加强法治宣传，弘扬法治精神，推进法律服务和援助工作。

　　三、进一步加强政府建设

　　积极适应经济发展、从严治党、从严治政和作风建设的新常态，以新的工作状态，更好地建设法治、为民、服务、效能、诚信、廉洁政府。

　　增强政治定力。深入学习贯彻习近平总书记系列重要讲话精神，认真落实省委“五句话”总要求，坚定政治立场，严守政治纪律，牢记宗旨使命，强化责任担当，扎实履职尽责，一切在大局下行动。毫不动摇地坚持以经济建设为中心，切实抓好发展这个第一要务，加快转变经济发展方式，实现有质量有效益可持续的发展。认真总结“十二五”规划完成情况，做好“十三五”规划编制工作，谋划好未来五年的发展蓝图，办好忻州的事情。

　　加快职能转变。处理好政府与市场的关系，充分发挥市场在资源配置中的决定性作用，更好发挥政府作用，最大限度减少政府对微观事务的管理，切实管好政府该管的事。坚持简政放权、放管结合、优化服务，深化行政审批制度改革，加快建设综合性政务服务平台，防止审批事项久拖不决。正确处理与基层的关系，充分调动县、乡、村工作的积极性。

　　严格依法行政。维护法律权威，严格依法办事，把政府工作全部纳入法治轨道，确保政府一切行为严格在法律和政策范围内活动。要尊法学法守法用法，依法全面履行职责，所有行政行为都要于法有据，任何政府部门都不得法外设权。坚持权力与义务、权力与责任、权力与程序、权力与监督的统一，不断增强运用法治思维和法治方式破解难题、推动工作的能力。积极推行政府法律顾问制度，认真做好行政复议工作。加强执法队伍建设，规范执法行为，完善执法程序，全面落实行政执法责任制。

　　自觉接受监督。自觉接受人大及其常委会的监督，主动接受政协的民主监督，加强行政监察和审计监督，重视社会和舆论监督。完善市级公共资源交易平台，打造公开透明的交易环境。引深“给人民汇报、请人民评议、向人民问计”政风行风活动，广泛听取群众的意见建议。积极探索移动互联网环境下政务公开的新途径，拓展政务公开的广度和深度。通过网站、短信、微信等多种渠道发布政务信息，自觉接受社会监督。进一步提升“忻州随手拍”微信问政平台和“忻州发布”政务微博运行水平，强化对群众反映问题的跟踪督办，积极回应社会关切。

　　持续改进作风。巩固党的群众路线教育实践活动和“学习讨论落实”活动成果，确保整改措施全部兑现。认真执行中央“八项规定”精神，自觉践行“三严三实”，严格落实国务院“约法三章”，坚持不懈纠正“四风”，在抓细抓常抓长上下功夫。牢固树立“先有预算后有支出”的意识，不断深化预算管理制度改革。2016年市本级和各县（市、区）社会保险基金预算向本级人大报告。清理规范重点支出同财政收支增幅或生产总值挂钩事项以及按人均经费标准安排的支出事项。建立结转结余资金定期清理机制，上一年预算的结转资金，应当在下一年用于结转项目支出。坚持倡俭治奢，严控“三公”经费，决不允许“吃空饷”，积极推进公务用车改革。

　　坚持从严治政。围绕实施“六权治本”，进一步加强制度体系建设，认真落实权力清单，加紧研究责任清单，积极探索负面清单，不断规范权力运行。对公共资金、公共资源、国有资产严加监管，坚决打掉寻租空间。不断完善政绩考核评价机制，教育引导政府工作人员“理政、谋政、勤政、善政、廉政”，对工作不力的要约谈诫勉，对懒政怠政的要公开曝光、坚决追究。严格落实“两个责任”，始终保持反腐败高压态势，用最坚决的态度减少腐败存量，用最果断的措施遏制腐败增量。

　　提升工作水平。坚持“执政为民、敢为人先、群策群力、滴水石穿”的忻州政府精神，以苦功夫、“笨办法”实现由量变到质变的跨越提升。进一步加强学习和调研，开阔眼界、完善思路、破解难题，提升干部队伍履职尽责的能力。进一步加快政府信息化建设，增强政府工作人员运用现代科技手段开展工作的能力。我们要始终把人民群众作为衣食父母，始终把为群众服务作为最大责任，以昂扬向上的精神状态和“功成不必在我、但求无愧我心”的工作追求，办好事关忻州发展的每一件事情。

　　各位代表，当前正是干事创业、加快发展的最好时期，经过反腐治乱，风气正了，环境好了，谁发展谁受益。这个时期，也是检验、识别干部的最好时期，谁敢担当、谁善担当，一目了然。让我们紧密团结在以习近平同志为总书记的党中央周围，在省委、省政府和市委的坚强领导下，同心同德，开拓进取，为开创弊革风清、富民强市新局面而努力奋斗！
